# Evidence of transgenerational effects on autism spectrum disorder using multigenerational space-time cluster detection

**DOI:** 10.1186/s12942-022-00313-4

**Published:** 2022-10-03

**Authors:** Rebecca Richards Steed, Amanda V. Bakian, Ken Robert Smith, Neng Wan, Simon Brewer, Richard Medina, James VanDerslice

**Affiliations:** 1 Department of Geography, University of Utah, 260 Central Campus Dr #4625, Salt Lake City, UT 84112 USA; 2 Department of Psychiatry, University of Utah, 501 Chipeta Way, Salt Lake City, UT 84108 USA; 3 Pedigree and Population Resources, Huntsman Cancer Institute, University of Utah, 2000 Circle of Hope Drive, Salt Lake City, UT 84112 USA; 4 Department of Family and Preventative Medicine, University of Utah, Salt Lake City, Utah USA; 5 Department of Geography, University of Utah, 537 W. 2900 S., Bountiful, UT 84010 USA

**Keywords:** Transgenerational, Space-time clusters, Autism spectrum disorder

## Abstract

**Background:**

Transgenerational epigenetic risks associated with complex health outcomes, such as autism spectrum disorder (ASD), have attracted increasing attention. Transgenerational environmental risk exposures with potential for epigenetic effects can be effectively identified using space-time clustering. Specifically applied to ancestors of individuals with disease outcomes, space-time clustering characterized for vulnerable developmental stages of growth can provide a measure of relative risk for disease outcomes in descendants.

**Objectives:**

(1) Identify space-time clusters of ancestors with a descendent with a clinical ASD diagnosis and matched controls. (2) Identify developmental windows of ancestors with the highest relative risk for ASD in descendants. (3) Identify how the relative risk may vary through the maternal or paternal line.

**Methods:**

Family pedigrees linked to residential locations of ASD cases in Utah have been used to identify space-time clusters of ancestors. Control family pedigrees of none-cases based on age and sex have been matched to cases 2:1. The data have been categorized by maternal or paternal lineage at birth, childhood, and adolescence. A total of 3957 children, both parents, and maternal and paternal grandparents were identified. Bernoulli space-time binomial relative risk (RR) scan statistic was used to identify clusters. Monte Carlo simulation was used for statistical significance testing.

**Results:**

Twenty statistically significant clusters were identified. Thirteen increased RR (> 1.0) space-time clusters were identified from the maternal and paternal lines at a p-value < 0.05. The paternal grandparents carry the greatest RR (2.86–2.96) during birth and childhood in the 1950’s–1960, which represent the smallest size clusters, and occur in urban areas. Additionally, seven statistically significant clusters with RR < 1 were relatively large in area, covering more rural areas of the state.

**Conclusion:**

This study has identified statistically significant space-time clusters during critical developmental windows that are associated with ASD risk in descendants. The geographic space and time clusters family pedigrees with over 3 + generations, which we refer to as a person’s *geographic legacy*, is a powerful tool for studying transgenerational effects that may be epigenetic in nature. Our novel use of space-time clustering can be applied to any disease where family pedigree data is available.

**Supplementary Information:**

The online version contains supplementary material available at 10.1186/s12942-022-00313-4.

## Introduction

Autism spectrum disorder (ASD) is a complex developmental syndrome that affects one in 44 children born in the United States as of 2021 according to the Centers for Disease Control and Prevention (CDC) [1], and Utah’s most recent published rate, one in 44 children in 2018 (https://www.cdc.gov/ncbddd/autism/addm-community-report/executive-summary.html) [2] . The disorder is characterized by neurodevelopmental characteristics and behaviors that vary in severity, impacting learning, communication, and social interactions [3–5].

The etiology of ASD is complex and includes both environmental and genetic factors [6–8]. As biotechnology has improved, the capacity for studying genetics and heritability has improved with the ability of genetic testing for ASD as more investigators have shown that ASD is highly heritable (heritability rates 0.61–0.73) [5, 9–12]. Through these efforts associations from maternal and paternal genetic variants show an increased risk for ASD [13, 14]. The genetic risks do not diminish findings that environmental factors that play a role in disease outcomes. Ambient air pollution exposure from polyaromatic hydrocarbons of roadway air pollutants, nitrogen dioxide, and particulate matter during vulnerable developmental windows of growth has been associated with increased risk of ASD and its severity [15–20]. Endocrine-disrupting chemicals, gestational infections, early life infections, and stress have also been found to contribute to the risk of ASD [21].

One intersection between the two etiologies of environment and genes can be epigenetics. Epigenes are small marks or switches on DNA that can silence or activate portions of DNA, essentially changing gene expression [22, 23]. Epigenetic mechanisms have been proposed as potential means by which environmental exposures in previous generations (2 + generations) might exert increased risks in future generations and induce increased levels of heritability [24, 25]. DNA methylation, histone modification, and RNA silencing are epigenetic mechanisms by which the environment acts on gene expression [26, 27]. Rett syndrome and Fragile X syndrome (FXS) are common comorbidities with ASD and show firsthand evidence of epigenetic methylation and non-binding RNA effects as mechanisms for ASD outcomes [28]. Exposures to nickel, cadmium, mercury, arsenic, pesticides, and other gases and particulates [29], all of which are considered environmental pollutants, have been found to impact epigenetics that contribute to disease outcomes across generations [28]. Epigenetic changes may originate during the ancestor’s (parents, grandparents, or previous ancestor) vulnerable developmental stages of growth, such as the prenatal and birth stage when developmental programming of organs is underway, and exposures occur [30]. Direct-contact exposure studies for the exposed generation have shown neurological impairment from certain exposures [31]. Perera et al. found that higher concentration exposures to incomplete fossil fuel combustion between gestation and 5 years of age resulted in statistically significantly lower IQ, and verbal scores [31].

Animal studies have confirmed transgenerational effects from environmental exposures [25]. Controlled laboratory settings simulating environmental pollution exposures from pesticides, fungicides, heavy metals, and petrochemicals have shown transgenerational effects in mice models [25, 32]. For the study of human subjects, challenges remain in testing the hypothesis that environmental exposures of ancestors’ affect ASD outcomes in progeny.

Space-time cluster analysis is one method used for exploratory research of environmental effects for hypothesis development. Among other things, it is used to align complex data and examine patterns of individuals with a disease suspected to be associated with an environmental exposure spatially and temporally [33, 34]. It can be extended as an approach for examining potential transgenerational effects of an environmental risk factor by identifying spatial-temporal patterns of grandparents and parents of individuals that have a health outcome associated with an environmental factor. The approach can be used to identify whether ancestors of ASD cases shared the same space and time, implying that there could be common factors (i.e., environment) elevating the risk of ASD among their descendants [35, 36]. Some diseases have been shown to originate during periods when growth and development are most susceptible to environmental stimuli (e.g., gestation when programming of specific organs is underway, or during childhood, adolescence, or preconception when rapid growth and development occur) [26, 30, 31, 37]. The approach can be further refined by focusing on these same developmental ages during ancestors’ lives in space-time cluster analyses. Using geographic residential data to investigate and identify the shared environmental space and time of parents and grandparents related to a child diagnosed with ASD could shed light on increased risks, vulnerable developmental windows that may be more susceptible to exposures and disease outcomes and provide evidence regarding whether there is a greater risk for disease of descendants associated with ancestral environmental exposures.

The aims of this study are to (1) Identify space–time clusters of parents and grandparents of children with a clinical ASD diagnosis and their matched controls. (2) Identify developmental windows of parents and grandparents with the highest relative risk for ASD in their children/grandchildren. (3) Identify how the relative risk may vary through the maternal or paternal line.

## Methods

### Study design

A retrospective space–time cluster analysis was used for the study design. Residential locations of parents and grandparents of clinically diagnosed ASD cases in Utah from 1989 to 2014 were compared to the residential locations of the ancestors of matched controls. This design is like other space-time cluster analyses of health outcomes, with the outcome defined as having a child/grandchild who has been clinically diagnosed with ASD. However, our design uses the ancestor generation(s) as the point of interest. The cluster analysis was carried out in six separate models for six types of ancestors: mothers, fathers, maternal grandmothers and grandfathers, and paternal grandmothers and grandfathers. For each of these groups, separate cluster analyses were applied for three periods of their lives, referred to as ‘vulnerable developmental windows’, representing windows of increased vulnerability to adverse effects of environmental stressors: birth/infancy (age 0–1 year), referred to as the “birth” window from this point on in the paper, childhood (age 2–11 years), and adolescence (age 12–17 years) [37] making it a total of 18 models for analysis.

### Inclusion criteria

**Fig. 1:**
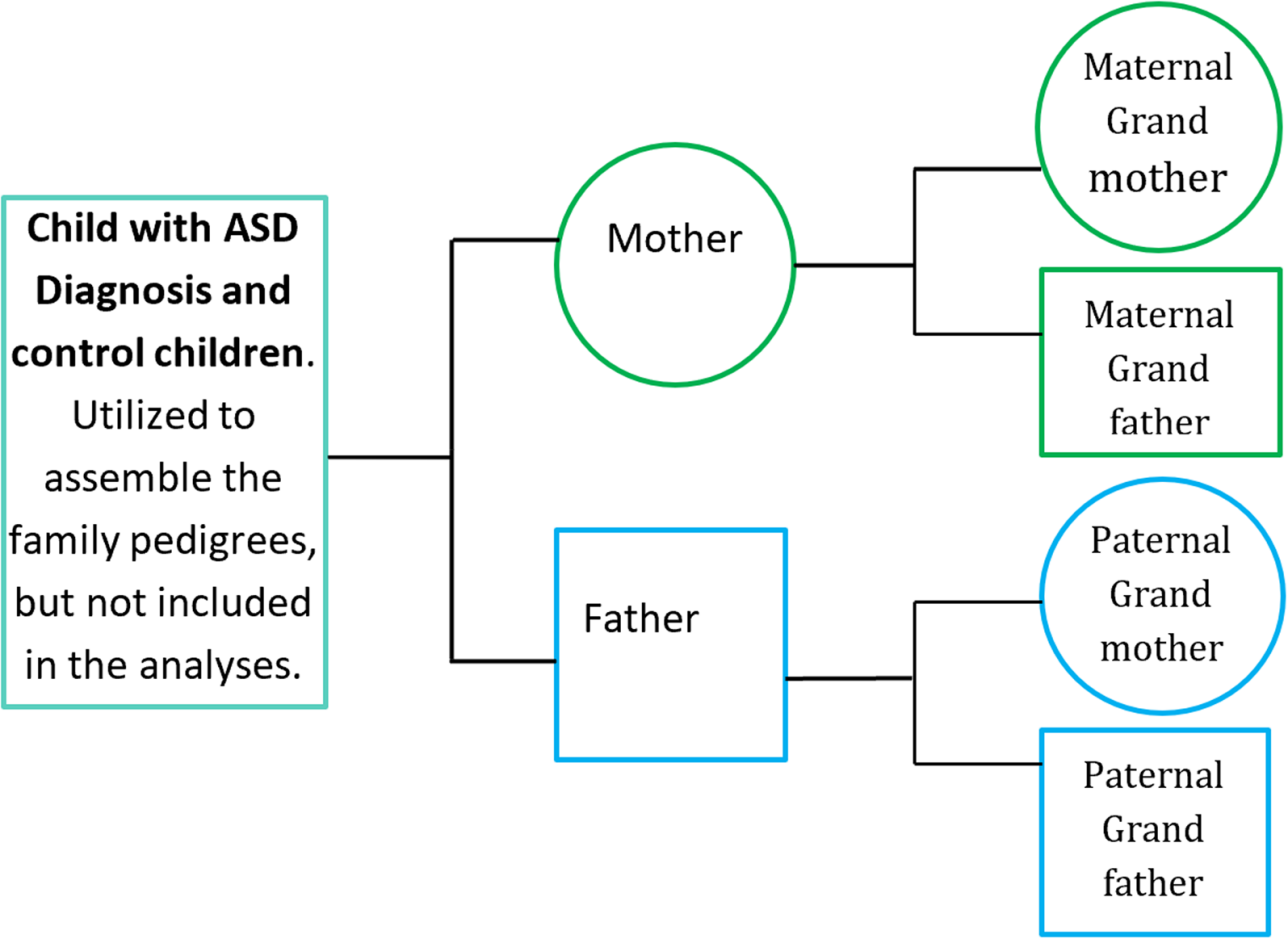
Family Pedigree Data Structure

For transgenerational research, the important exposure group is the ancestors of individuals with an ASD diagnosis. The individuals with ASD were not used in the modeling and analysis. They were used only to find their ancestors to build their family pedigrees. Ancestors of the ASD cases were defined as the eligible parents/grandparents of individuals with a clinical diagnosis of ASD with a birth year between 1989 and 2014. The Utah Registry of Autism and Developmental Disabilities (URADD) was the source of the ASD individuals. URADD classifies ASD individuals using a spectrum of diagnostic billing codes (ICD 9 29,900, 29,901, 29,910, 29,911, 29,980, 29,981, 29,990, 29,991 and ICD 10 F84.0, F84.2, F84.3, F84.5, F84.8, F84.9). The parent/grandparent cases used in the analysis were linked to the URADD individuals using the Utah Population Database (UPDB), an extensive multi-database repository that can generate family pedigrees from administrative data. Non-case ancestors were identified in the UPDB and defined as parents/grandparents of the randomly selected children matched on age and sex of case children born between 1989 and 2014. These parents/grandparents were included in the study if they met the following criteria: (1) the diagnosed child was the first reported case of ASD in the family according to the records in URADD linked to the pedigree data in UPDB; and (2) the parent/grandparent had a Utah birth certificate, a Utah medical record during childhood (age 1–17), and/or a record of a Utah driver’s license. The ASD child birth years in the data range from 1989 to 2014, parent birth years range from 1949 to 1995, and grandparent birth years range from 1929 to 1977 (Fig. 1). Family Pedigree and Data Structure is a graphic that gives a visual overview of our family case and none-case selection structure.

### Residential history

Residential locations for the parents and grandparents of the ASD case and control children were obtained from the UPDB. Residential locations came from several administrative sources from the UPDB. Specifically, birth certificates were used to locate parent’s and grandparent’s residences at their time of birth. Medical records, including inpatient records, All Payers Claims, and Emergency Department records were used to place the parents and grandparents in childhood at residential locations. Medical records and driver’s license records were used to identify the residential location of parents and grandparents during adolescence.

### Cluster analysis

A spatial scan statistic Bernoulli space-time binomial distribution model was used to identify space-time clusters for each group of ancestors at each of the three exposure windows. The Bernoulli model is a discrete binomial distribution scan statistic that tests for binary outcomes; ‘case’ or ‘non-case’ present in a population at any given place and time using varying size elliptical cylinder windows [38–40]. One of the advantages of using the Bernoulli distribution is its sensitivity to point-level location data in case and non-case populations. The statistical significance of each cluster (p < 0.05) was estimated using Monte Carlo simulation with 999 permutations representing the random placement of cases. A Bonferroni-corrected p-value was calculated to control for Type I error [41]. A scan statistic then computed and compared the maximum likelihood ratio from the dataset to randomly generated permutation datasets with the assumption there are no clusters. The maximum likelihood ratio test was used to identify the most likely cluster to have occurred in the analysis. A relative risk value for each cluster was then calculated by using the estimated risk within each space-time cluster divided by the estimated risk outside of the cluster [38].

Many case and non-case ancestors did not change residential locations over time, particularly between birth and childhood time periods. As such, a cluster arising from a factor present during the birth window may also be detected for the childhood exposure window of the parent or grandparent. To assess whether identified clusters within the same ancestor group were distinct or comprised of the same individuals, we determined the number of subjects in a cluster who also fall within the space of another cluster, as shown in the Additional file: 1 Table S1. Overlap Analysis Results.

Residential locations were tested for spatial autocorrelation to identify areas that might get over-predicted in the binomial distribution cluster analyses. An over-prediction can occur if location points are dense in any given area [42]. As expected, spatial autocorrelation is present for one area with the highest population density in the state. However, no spatial clusters were identified for our cluster areas.

## Results

The study used 3957 individual ASD cases to link 7914 parents and 15,828 grandparents over space and time, matched 2:1 at the case level for age and sex (Table 1). Number of Subjects and Ranges of Birth Years by Relation provides breakdown of our general data and what we had available to use.

**Table 1:** Number of subjects and Ranges of Birth Years by Relation

Family relation (birth year range)	Percent linked to a residential location (%)	Total subjects	Infant	Childhood	Adolescence
Mother (1948–1998)	91	Case, n = 3957	3957	2734	2993
		Non case, n = 7914	7914	5468	5986
Father (1925–1997)	93.5	Case, n = 3957	3957	3994	2374
		Non-case, n = 7914	7914	7914	4748
Maternal grandmother (1903–1982)	92.5	Case, n = 3924	1705	2861	783
		Non-case, n = 7848	3410	5722	1566
Maternal grandfather (1873–1975)	92.5	Case, n = 3774	3584	3289	929
		Non-case, n = 7548	7168	6578	1858
Paternal grandmother (1907–1977)	90.5	Case, n = 3924	1644	2855	1119
		Non-case, n = 7848	3288	5710	2238
Paternal grandfather (1897–1977)	91.3	Case, n = 3795	1644	3336	1063
		Non-case, n = 7590	3288	6672	2126

**Fig. 2:**
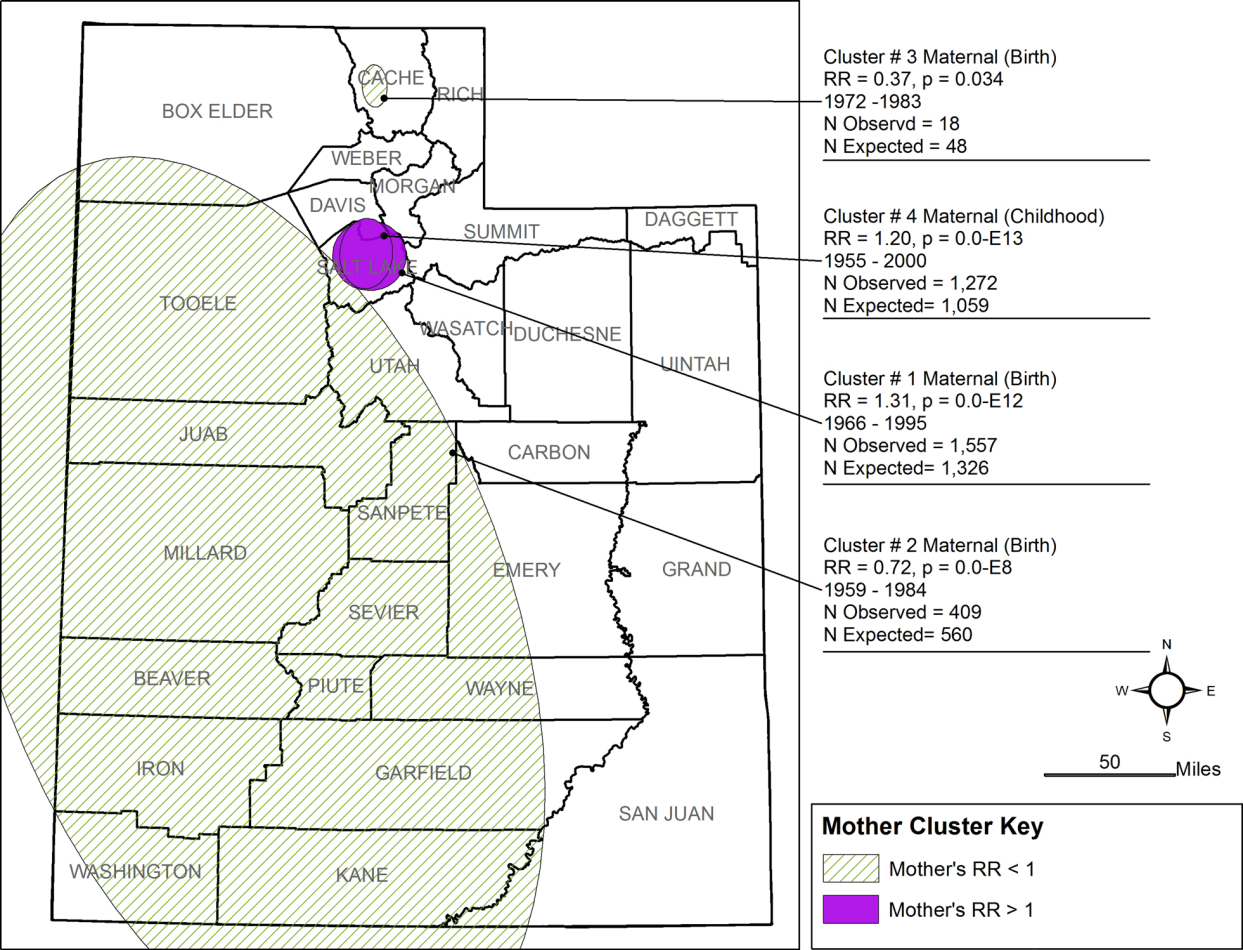
Clusters of Residential Locations of Mothers of ASD Cases in Utah

**Fig. 3:**
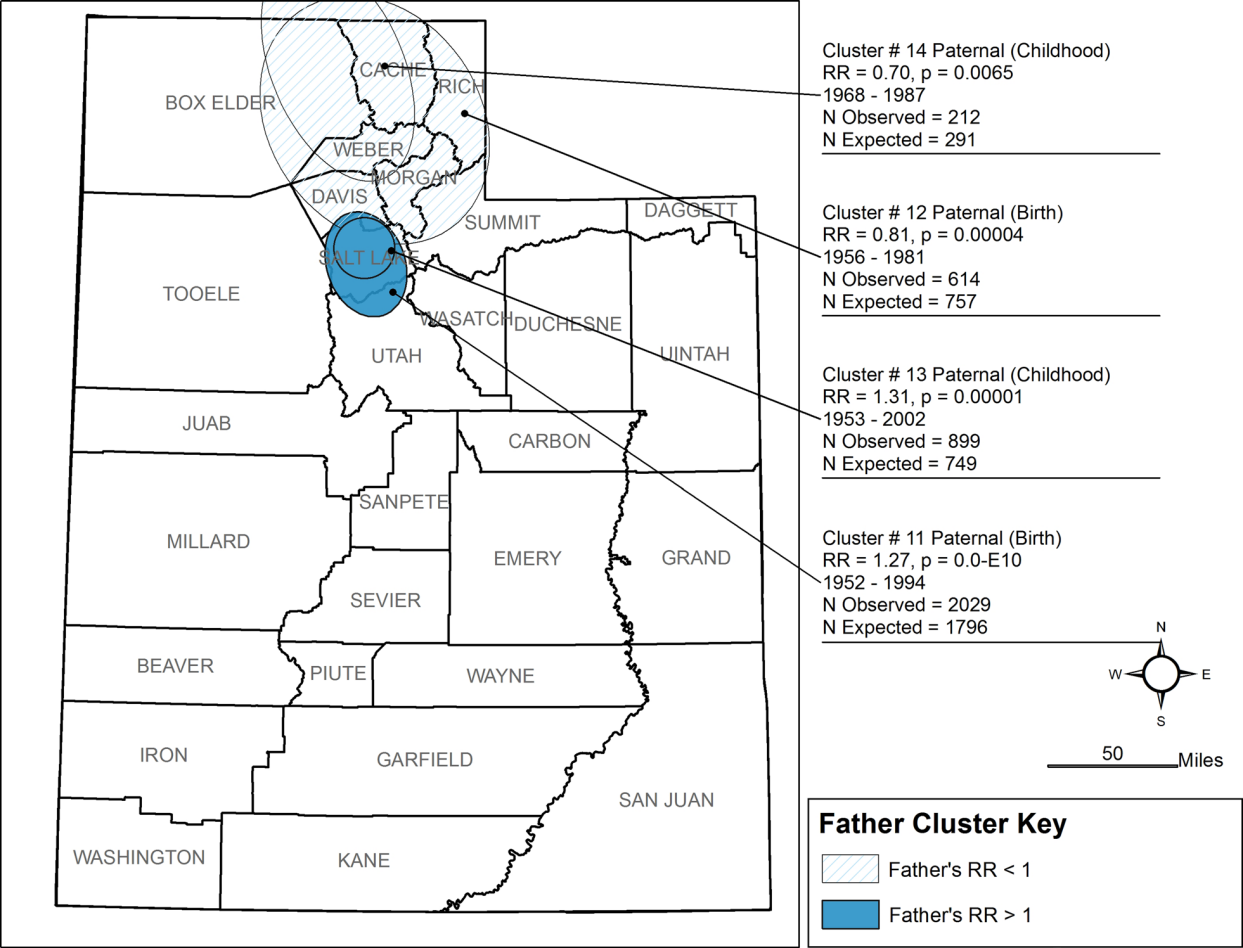
Clusters of Residential Locations of Fathers of ASD Cases in Utah

**Fig. 4:**
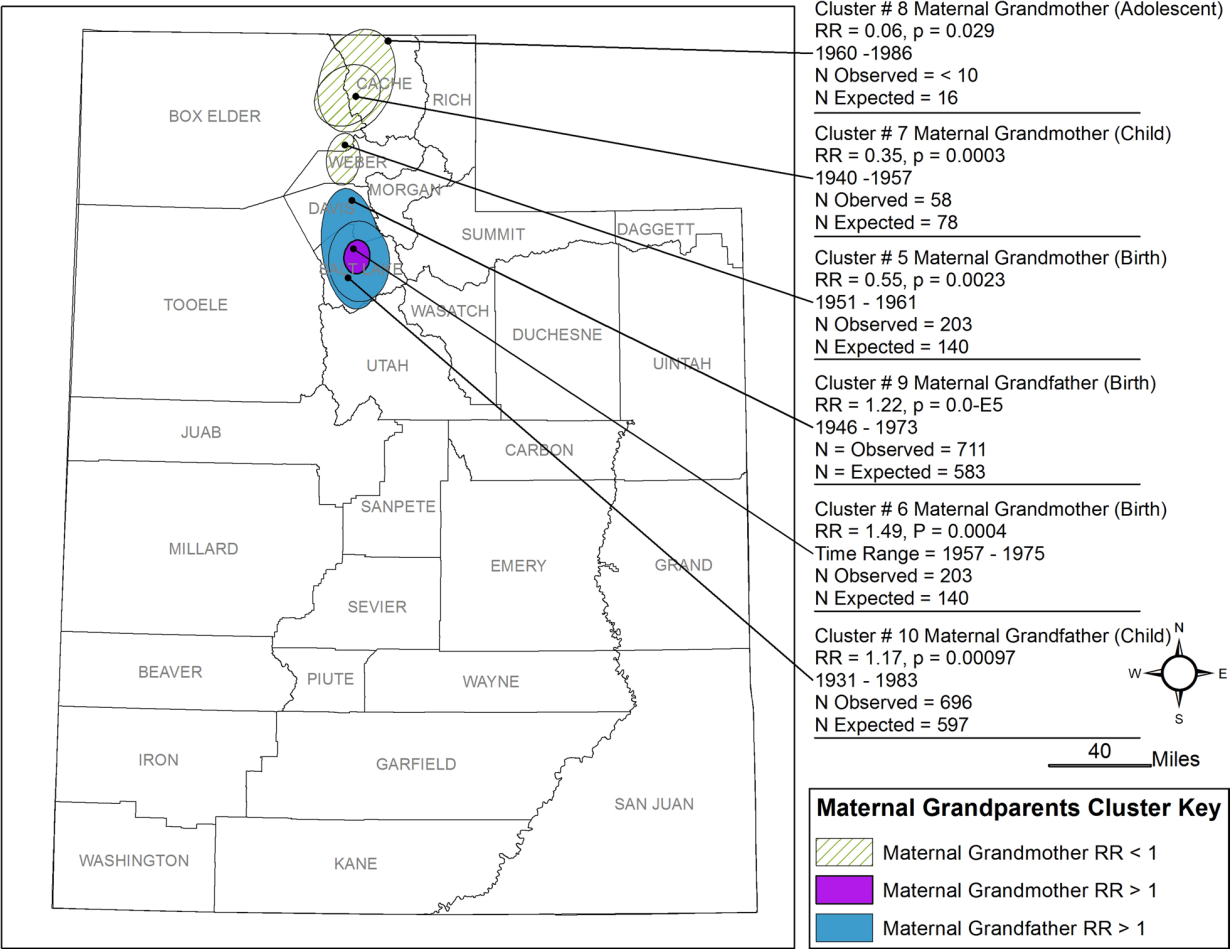
Clusters of Residential Locations of Maternal Grandparents of ASD Cases in Utah

**Fig. 5:**
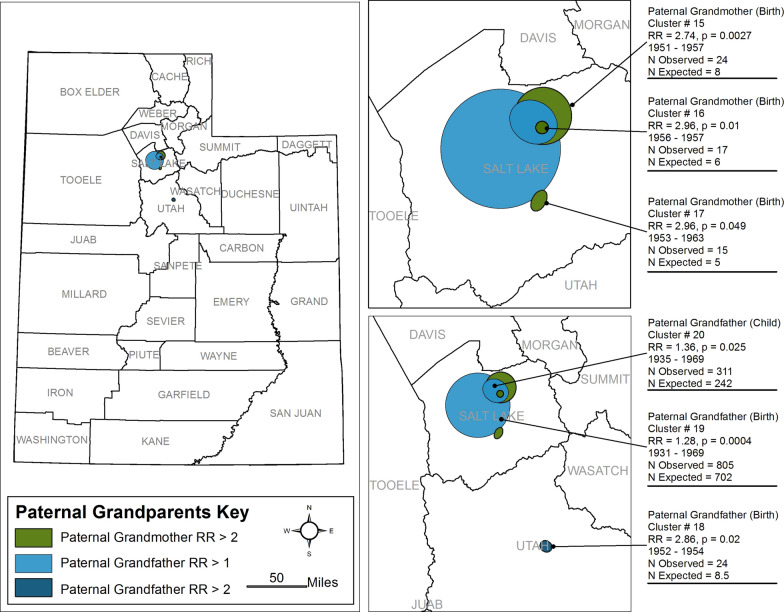
Clusters of Residential Locations of Paternal Grandparents of ASD Cases in Utah

Our analysis found 64 space-time clusters among case families, 20 of which are statistically significant (< 0.05). The 20 clusters occurred between 1930 and 2002, seven with RR < 1.0 (See Figs. 2, 3, 4, 5, Table 2). Seventeen had p-values < 0.01, and three had p-values 0.01 < p < 0.05. All ancestor types of ‘parent’ or ‘grandparent’ have birth and childhood clusters. Only one cluster was associated with the Adolescent window (Maternal Grandmother, RR = 0.06) of 13 clusters with RR > 1 (range = 1.27–2.96). Nine of the 13 clusters are among grandparents four are among parents. Eleven of the thirteen clusters range in the narrow time window of 1946–1960. Parent clusters are larger in area size (1633–4248 km^2^) than the grandparent birth and childhood residential location clusters and have RR > 1.2. Three clusters among maternal grandparents with RR between 1.0 and 2.0 occurred in predominantly urban areas.

**Table 2:** Ancestor Space-time Cluster Results

Relative	Developmental window	Cluster number	Time range	Observed/expected	RR	P-value	Bonferroni correction	Area km^2^	Percent street location (%)	Cohort effect	Census rural/urban classifications
Mother	Birth	1	1966–1995	1557/1326.20	1.31	0.0001	1.0E−7	2286	93	542 → Cluster 4	Urban
		2	1959–1984	409/560	0.70	0.0001	1.0E−7	122	93	0	Rural
		3	1972–1983	18/48	0.37	0.034	3.4E−5		93	0	Rural
	Childhood	4	1955–2000	1272/1059	1.2	0.0001	4.0E−7	1633	93	542 ← Cluster 1	Urban
Maternal grandmother	Birth	5	1957–1975	203/140	1.49	0.0004	1.9E−5	226	91	0	Rural
		6	1951–1961	68/119	0.55	0.0023	2.3E−6	408	91	0	Urban
	Childhood	7	1940–1957	58/78	0.34	0.0003	3.0E−7	482	91	53 → Cluster 8	Rural
	Adolescence	8	1960–1989	1/16	0.06	0.029	2.9E−5	940	100	53 ← Cluster 7	Rural
Maternal grandfather	Birth	9	1946–1973	711/583	1.36	0.0001	1.0E−7	3247	91	3 → Cluster 10	Rural/Urban
	Childhood	10	1931–1983	696/597	1.32	0.0009	9.0E−7	2336	89	3 ← Cluster 9	Urban
Father	Birth	11	1952–1994	2029/1796	1.27	0.0001	1.0E−7	4248	93	431 → Cluster 13	Urban
		12	1956–1981	614/757	0.77	0.00004	4.0E−8	25,943	93	10 → Cluster 14	Rural
	Childhood	13	1953–2002	899/749	1.31	0.0000	1.0E−7	1661	92	431 ← Cluster 11	Urban
		14	1968–1987	212/291	0.70	0.0065	6.5E−6	14,733	92	10 ← Cluster 12	Rural
Paternal grandmother	Birth	15	1951–1957	24/8	2.74	0.002	2.0E−6	24	91	0	Urban
		16	1956–1957	17/5.7	2.96	0.01	1.0E−6	8	91	91%	Urban
		17	1953–1963	15/5.1	2.96	0.049	4.9E−5	14	91	0	Urban
		18	1952–1954	24/8.5	2.86	0.002	2.0E−6	132	91	0	Urban
		19	1931–1969	805/702	1.28	0.004	4.0E−6	681	91	0	Urban
Paternal grandfather	Childhood	20	1935–1969	311/242	1.36	0.025	2.5E−5	98	89	0	Urban

Statistical significance of each cluster was measured at p < 0.05 using Monte Carlo simulation

A cohort effect was observed between two cluster pairs. Spatially overlapping clusters of birth #1 cluster share 42.6% membership with childhood #4 cluster from the maternal lineage. Paternal birth cluster #11 shares 48% membership with childhood cluster #13. The overlapping membership signifies individuals that did not move between vulnerable developmental windows and contributed to statistically significant space-time clustering for the next vulnerable developmental window from the same lineage group. Though most of the clusters overlap in space and/or time, very small, or no shared membership was found between developmental window clusters and lineage between other statistically significant clusters (see Table 2).

Of the seven clusters with lower risk (RR < 1), five are from the maternal side of the family, with four divided between the mother and father. All are in rural areas, with six of the seven occurring in the northern part of the state (Figs. 2, 3, 4). Compared to these clusters, clusters with RR > 1–1.49 are larger (area = 98–3247 km^2^), longer in duration (18 to 52 years), are predominantly urban, and are both maternal and paternal.

Approximately 9% of the address records were associated with PO Boxes in rural areas. We used a sensitivity analysis to assess potential bias in these locations. We moved cluster points 10 m in random directions and re-ran the space–time cluster analysis. The clusters remained, although rural clusters lost their statistical significance.

## Discussion

To our knowledge, this is the first transgenerational space-time cluster analysis to study ASD health outcomes in progeny. Our study has used space-time cluster analysis as a novel means of exploring transgenerational risk using residential locations and time windows of parents and grandparents of ASD case children during vulnerable developmental windows of exposure. We identified 20 statistically significant space-time clusters of residential locations of parents and grandparents of varying size, duration, and varying levels of risk. The identified clusters are diverse in location, with a general trend toward spatially smaller sized clusters located in more urban areas for their time durations, and larger sized clusters with a mixed composition of largely rural areas with some urban areas based on Census designations from 1960 to 2000 for applicable clusters [43]. Census records with designations predating 1960 for Utah are not available. Therefore, decisions about rurality and urbanicity were made based on 1960 classifications, with the presumption that rural areas would still be rural in 1960, and urban areas predating 1960 would still be urban in 1960 [43].

It has been posited that the urban–rural trend in our results is a consequence of reporting bias, where cases are less likely to be reported in rural locations [44]. However, for our analysis it is the ancestors used in statistical calculations, not the ASD case children themselves. Additionally, the urban–rural trend we observed in our results has also been observed in Swedish ASD studies [45].

Transgenerational research requires extended family pedigree analysis with the ability to identify exposures of ancestral generations that are no longer present in affected generations [25]. This study was able to take the initial steps in accomplishing this in a novel way with the use of space-time cluster analysis of ASD using grandparent and parents of ASD case’s residential histories in early life. Space-time clusters provide the location and time of a shared space, which can be used to generate hypotheses regarding the underlying factors associated with those places and times that might be the explanatory, putative, and underlying factors that produced the health outcome in progeny. The putative factors could be any condition or conditions present at those locations and time periods where individuals were more likely to share conditions including environmental contaminants, including endocrine-disrupting chemicals, dietary patterns, and nutritional deficiencies, or heightened psychological stress resulting from social or economic conditions [46]. Effects may be dependent on maternal or paternal inheritance and transmission. Paternal grandparent nutrition in childhood, for instance, directly impacts sex-specific health outcomes in grandchildren [47]. Clusters from our analysis with the highest RRs are from the paternal grandmother and grandfathers, occurring at a time when it was common to use pesticides containing endocrine-disrupting chemicals and petrochemicals in the home [48]. DDT was one of the more commonly used pesticides and has well-documented epigenetic transgenerational effects [25, 49, 50].

We have several interesting findings. The Mother and Father clusters (see Figs. 2 and 3) generated similar results in the number of clusters, p-value, and time span except for the large, detected cluster encompassing the southwest counties. Maternal Grandmother clusters results show the same cluster pattern as the mother and father clusters. To determine if residential location remained the same between generations, an overlap analysis was conducted identifying the family member and shared residential location between clusters and ancestor generation. We found that less than 5% remained in the same residential location as the ancestor group.

Arguably, our most interesting findings are the paternal grandparent clusters with RR > 2.74. These clusters are at a sub-city scale in size and are highly compact in time and space. The clusters occurred in urban settings of their time, suggesting that the underlying factors may be associated more generally with the urban environment than with specific unique point sources of contamination. This is not to say that diagnostic bias in rural and urban areas has not occurred. Family members are also more likely to share the same space-time, so clusters may reflect the clustering of genetic predispositions from clustering of family members. However, grandparents share fewer genes with grandchildren which suggests our paternal grandmother clusters and paternal cluster could be non-genetic factors. Because we have moved forward in time from grandparent-to-parent in our space-time cluster methodologies, it has not been possible to determine the level of relatedness between ASD proband children, as they have not been the focus of the study outside of pedigree building. This creates an opportunity to study the specific question of relatedness and genetic similarity of clusters in future work.

Both genetic and epigenetic changes may be possible mechanisms to explain how the environment can impact an ancestor, which may be transferred transgenerationally to a descendent. Transgenerational health outcomes from environmental exposures have been well established in animal studies where multiple generations of animals live and are carefully observed for changes in health patterns [25, 51, 52]. The same types of transgenerational observations in human populations are more difficult to observe as family location data with disease outcomes are not readily available, but they still exist. A study investigating persistent ionizing radiation exposure in grandparent generations, for example, found an increased incidence of low bone density in their young adult grandchildren [53]. Another notable example is the investigation into grandparent exposure to dioxin TCDD, an herbicide used in the Vietnam War as a chemical agent. Researchers found mutations that occurred in spermiogenesis that impacted the health of their progeny, including leukemias [54, 55]. Tabaco smoke exposure is well studied in humans and transgenerational effects are recognized [56].

## Strengths and limitations

### Strengths

The study has several strengths, most relating to the high data integrity and the richness of the records, allowing investigators to place individuals in pedigrees, and throughout space and time. The dataset allows us to link family members over space and time at different vulnerable developmental windows and has been key to identifying space-time clustering. The rich location data within the records provide sufficient information to include thousands of records. As a result, the analysis was based on a large number of ancestors of ASD cases starting with a very complete case ascertainment for case children/grandchildren born in Utah from 1989–2014 (n = 3957).

Using the unique linked pedigree and vast administrative data in the UPDB we were able to generate a large dataset of residential locations for both parents and grandparents at three critical developmental windows with a high level of completeness. The dates and residential locations used are based on a consistent and reliable source of information—birth certificates and medical records, with the majority of our addresses being home addresses (> 90%).

Additionally, we used only clinically diagnosed ASD case children to create our family pedigrees. While keeping our ASD cases defined by clinical diagnosis, we used a broad statewide dataset, as opposed to limiting the study to a specific county or regional jurisdiction. This decision was made to ensure the study included both rural and urban residential locations over space and time.

The study has several data limitations. The study includes Utah children only. The URADD dataset is specific to Utah and does not include any other person outside of the state. We omitted the sex of the case children when compiling the pedigrees. This will be completed for a future study when permissions are granted to include case children in the analysis. We did not have any information relating to adoption. Family pedigrees were generated based on birth certificate information. If a non-biological person was on a birth certificate, we would not have information indicating they are adopted or had adoptive parents. We lacked information on the relatedness of grandparents to each other. This can be addressed in future studies. Also, we did not have complete residential histories. Our study was limited to information in administrative records from URADD and UPDB. If a person moved and did not generate a record for that time we would not be aware of such moves. This omission, however, would likely serve to generate conservative (i.e., RR’s biased toward 1) estimates of risk.

## Future studies

Our understanding of possible transgenerational factors affecting the risk of ASD could be improved by other cluster analyses to corroborate these findings. In addition, analytical studies are needed to assess the relationships between environmental factors experienced by parents and grandparents and the likelihood that their descendent have ASD. Our cluster analysis results can be used to further study exposures of the temporal ranges identified, and at around the cluster locations identified which in so doing helps bridge the gap between transgenerational exposures and heritable health outcomes. Additionally, with our current results and family pedigrees we have compiled, we can further investigate the rural and urban locations of ASD case children and their location-where-diagnosed to determine if they maintained a rural or urban residence that matches their ancestor identified in clusters.

Given that little is known, and very little work has been done regarding transgenerational disease inheritance from environmental exposures, our space-time cluster study, using individual records, will add to the growing body of knowledge regarding this subject. The framework used and presented in this paper can be employed by any study looking for health effects from unmeasured ancestral space-time exposures.

## Conclusion

Our study findings indicate that specific shared time and space of ancestors is associated with an increased likelihood of an ASD diagnosis in decedents. At this point, these results do not provide information that can be directly used to identify individuals at higher risk or provide other direct benefits to individuals with ASD. The results to provide some evidence of transgenerational risks and lays the foundation for future research to identify risk factors that can lead to increased risks of ASD and other adverse health outcomes across generations. We are currently examining the associations between a variety of environmental conditions during critical exposure windows of parents and grandparents and the risk of ASD in their progeny. Identifying and addressing such risk factors may lead to reduced risks for future generations. These implications are broad and need further investigating, but the time and space of interest and the family pedigrees of interest have been identified and can be studied further.”

Our strongest signal identified is from the paternal grandparent’s birth and childhood vulnerable developmental windows. Subdividing the data by maternal and paternal lineage revealed surprising results of the paternal grandparent clusters carrying the highest relative risk at statistically significant levels (P < 0.05), which is a possible signal for a transgenerational effect from birth and childhood exposures.

## Supplementary Information

**Additional file 1: Table S1.** Overlap Analysis Results.

## Data Availability

The data that support the findings of this study are available from URADD and UPDB but restrictions apply to the availability of these data, which were used under license for the current study, and so are not publicly available. Data are however available from the authors upon reasonable request and with permission of URADD and UPDB.

## Abbreviations

ASD: Autism spectrum disorder
UPDB: Utah population database
URADD: Utah registry of autism and developmental disabilities
