# Supplementary material for: Evidence of transgenerational effects on autism spectrum disorder using multigenerational space-time cluster detection
Source: Int J Health Geogr. 2022 Oct 3;21:13. doi: 10.1186/s12942-022-00313-4 (PMC9531495; doi:10.1186/s12942-022-00313-4)
Supplement: Supplementary file 1 — Additional file 1: Table S1. Overlap Analysis Results. [file 12942_2022_313_MOESM1_ESM.docx]

## Table S1. Overlap Analysis Results

| **Lineage** | **Cluster** | **Count Overlap** | | **Into** |
| --- | --- | --- | --- | --- |
| Maternal | 1 | 11 | | 2 |
| Maternal | 1 | 1077 | | 3 |
| Maternal | 2 | 11 | | 1 |
|  |  |  | |  |
| Maternal | 2 | 22 | | 3 |
| Maternal | 3 | 1077 | | 1 |
| Maternal | 3 | 22 | | 2 |
| Maternal GM | 5 | 5 | | 6 |
| Maternal GM | 6 | 5 | | 5 |
| Maternal GM | 6 | 7 | | 7 |
| Maternal GM | 7 | 7 | | 6 |
| Maternal GF | 8 | 418 | | 9 |
| Maternal GF | 9 | 418 | | 8 |
|  |  |  |  | |
| Paternal | 10 | 0 | 0 | |
| Paternal | 11 | 0 | 0 | |
| Paternal | 12 | 0 | 0 | |
| Paternal | 13 | 0 | 0 | |
| Paternal GM | 14 | 15 | 15 | |
| Paternal GM | 15 | 15 | 14 | |
| Paternal GM | 16 | 1 | 14 | |
| Paternal GM | 14 | 1 | 16 | |
| Paternal GM | 17 | 0 | 0 | |
| Paternal GF | 18 | 3 | 20 | |
| Paternal GF | 20 | 3 | 18 | |
| Paternal GF | 19 | 311 | 20 | |
| Paternal GF | 20 | 311 | 19 | |
